# Keystone Veterinary Medical Association

**Published:** 1891-11

**Authors:** W. S. Kooker

**Affiliations:** Secretary


*Keystone Veterinary Medical Association.*—The regular meeting of the Keystone Veterinary Medical Association was held at the College of Physicians, Philadelphia, October 3rd. The meeting was called to order by the president, W. Horace Hoskins. There were present: Hoskins, J. B. Rayner, Kooker, Lintz, Cullen, Webster, Eves, Werntz, and Schreiber; also as visitors, Drs. Senseman, Gadsden, McDowell, and Benjamin Lee, Secretary of the Board of Health. The secretary read a communication from Dr. A. Liautard acknowledging receipt of invitation and regreting his inability to be present on account of the opening of the American Veterinary College.

The president read the following communication received by morning mail:

Dear Doctor: I will certainly appear to-day at the College of Physicians before the Keystone to read my paper. But I will not say very much, on account of the controversy and very entirely opposite views and experiences on this subject. But it might lead to interesting discussion. Yours,

H. P. Formad.

And the following, received by evening mail:

Dear Doctor Hoskins: My brother is in distress to-day. He left for New York at 3 P. M., this afternoon to attend to a criminal case of great interest and which had to be attended to at once. He even did not have time to notify you personally, as his departure was sudden, and asked me to wait upon you. Brother suggests some one else open the discussion on the question at issue to-night, which no doubt will lead to an interesting discussion. He is very sorry not to be able to participate in it.

Very respectfully, Robert Formad.

The secretary read the following communication:

1008 N. 6th Street, Philadelphia.

Dr. W. S. Kooker,
Secretary of the Keystone Veterinary Medical Association.

I hereby present my resignation as a member of the Keystone Veterinary Medical Association. Respectfully,

Robert Formad, V. M. D.

Philadelphia, October 1, 1891.

Dr. Lintz proposed the name of John MacFayden for associa temember. The last two communications were referred to the Board of Trustees for action. The president said that he was extremely sorry for the absence of Dr. Formad, as the subject of the transmission of tuberculosis by milk, was one of great interest to the sanitarian, whether human or veterinary practitioner. Dr. Gadsden was called upon. He said that in his own practice he had seen many cases, which point to the danger of using infected milk. He gave the case of a cow affected with the disease, which he ordered destroyed ; but it was not. The owner's wife used the milk of the cow, drinking it warm ; and, later, contracted tuberculosis, frcm which she died.

Dr. Werntz reported a case of a healthy calf, which was experimentally fed at the udder of a tuberculous cow, and contracted the disease ; and other cases in which healthy pigs were fed on tuberculous milk and contracted the disease. There was a family living in West Philadelphia, consisting of a father, mother, and eight children ; the father an exceptionally strong, healthy man from a family of long lives and free from any tubercular taint ; the mother had no knowledge of any of her family having died from any tuberculous trouble—all were healthy people. Six of her children were raised by the breast and all the very type of physical strength. The other two were raised by the bottle (on account of the mother having in each case a gathered breast). These two died with consumption. The inference to be drawn in these cases is, that the germs were taken into the system by the milk from tubercular cows.

Dr. Webster stated that there was a great amount of tuberculosis in his practice. In one dairy they fed some of the milk to a pen of pigs, which produced the disease in them. One of the pigs was killed and the tubercular lungs sent to him for examination, and a part of the lungs was eaten by a cat, weighing not less than twenty pounds. The cat is now in the very last stages of consumption.

Dr. Eves states that about Wilmington, there is a great deal of the disease among the cattle, the milk of which is consumed by the citizens of Wilmington. Drs. McDowell and Cullen said that their experience agreed with that of the previous speakers. Dr. Benjamin Lee, Secretary of the Board of Health of Philadelphia, said that he was glad to be present and to see the interest taken in the subject. Go on agitating and investigating the subject and his board would give all the aid that lays in its power. There is no doubt in his mind that many of the diseases that are not traceable are due to the transmissibility of the germs from diseased meats or milk. What we want is greater interest to be taken in the subject by the veterinarian and physician. If the medical practitioners of both branches would do their duty we could get legislation to greatly decrease disease and epidemics. You are all aware of the efforts made by the Board of Health to prevent the pollution of the streams of the State, and our defeat by our learned Legislature, due, in part, to the apathy of the medical profession and largely to the money of the manufacturers, dyers and butchers. Dr. Schreiber, Milk Inspector for Philadelphia, was of the opinion that tuberculosis is transmissable not only from animal to animal but also to the human subject. The best and surest way to remedy the evil is to destroy the animals affected.

Dr. Eves reported the following cases: He was called on June 16, 1889, to visit a cow suffering—as the owner stated—with a mysterious disease; having had seven to die within two or three weeks, this one making the eighth. The herd consisted of nine cows and one calf. He found No. 8 very weak—staggered when made to walk. Her posterior limbs were apparently partially paralyzed; slight flow of saliva; eyes of an anxious, haggard appearance; slight protrusion; diarrhœa; painful tenesmus; symptoms given of frequent licking and biting at the feet and lower part of legs (posterior); appetite impaired. No trouble given as to drinking. Inclined to be dull but at times uneasy. Pulse weak and accelerated. Temperature normal. History of having been sick for several days and growing weaker. The other seven were held in about the same way, excepting one Jersey cow which was inclined to be cross but not violent. The owner placed a great deal of stress upon the fact that they were continually biting and licking their feet and legs. He would not allow me to destroy her, but was to inform me of her death. Searched the pasture; found nothing unusual. Cows had been running on pasture as usual all spring. On July 3d, held post mortem and found No. 9 sick also; destroyed her, and found both presenting about the same lesions. Rumen contained a quantity of grass. Omasum was in a state of dry impaction (very dry). Posterior to this, intestines and fourth stomach were empty. Intestines manifested lesions of diarrhœa (no enteritis). The liver tended to be rather friable. In fact, no marked lesions excepting the impaction of third stomach which was complete in both cases.

The owner asked me this time to give him the symptoms of hydrophobia in the dog, which I did. He then told me that his dog certainly had the disease and that he had seen him chasing and biting the cows several weeks previously. He acted so strangely that he shot him; but at time thought nothing of his chasing the cows excepting that it was unusual for him to do so. I did not make a diagnosis; in fact, I was at loss to know what disease I was dealing with. On July 4th, the next day, I was called in another direction—about eleven miles from the first place—to see some cows. I found two cows with precisely the same symptoms as the others I had visited, with the history of several having died within two weeks. These cows were not violent—I could handle them as the others; but they seemed to be a little wild. I learned the history of two dogs having been seen biting the cows about five weeks previous to my visit. The cows were bitten, and bitten considerably; but the bites were all healed at the time of my visit. Did not get a chance to hold post mortem.

On July 17th was called by another party to see a cow in the same district. Cow showing same symptoms, no history of any dogs; but his neighbor, about half a mile distant, had lost nearly all his cows, and he had shot a dog in the act of biting them. I drove over and received a complete history from the man that shot the dog. The symptoms were precisely the same as those given previously, and every cow that was bitten died. His brother directly opposite had also lost a number of cows; the same dogs were seen among his cows and had bitten them.

These cows had all been treated by a veterinary surgeon, but not one had yielded to treatment. The dog was a collie and belonged to a farmer about three miles distant. His partner (the other dog) escaped, but was shot some time afterwards by his owner on account of acting in a peculiar manner. About a week after this another man from nearly the same neighborhood, only one mile from cases No. 2, called me to see a cow. I found her down with the same symptoms, but much wilder. Her eyes were rolling, her head swinging, and she was bellowing and frothing at the mouth. He had had five others die and one of them became very violent and was shot while in the act of breaking everything to pieces. The same dogs were seen among his cows. I came to the conclusion after seeing cases No. 2, that I was dealing with Rabies and after getting the history later, I am confirmed of the diagnosis of Rabies, as these farmers are all reliable men and I believe their cows were bitten by these dogs, as they state, and the same dogs, with the exception of cases No. 1, visited each herd that was affected. I at first thought of Texas fever, but the post mortem changed my opinion immediately. About fifty cows in all were affected and died.

Dr. Hoskins reported the following case: The subject of this somewhat unusual and unique case, was a large Maltese cat about two years old. Was injured on March 9th from a flobert rifle; with bullet marks at both inner and external canthi of right eye. The cat ordered to my infirmary on the 10th. Treated antiseptically, in conjunction with anodynes locally, until the 26th, when I considered extirpation of the entire eye the best treatment, which was done. On the 5th of April the animal was sent home, with the entire healing process apparently completed. On June 8th my attention was again called to the cat, and I found the orbital cavity partially filled with a catarrhal discharge. A mild astringent wash gave the necessary relief in a few day. On August 25th, I was summoned to attend him for lameness. A thorough examination of the right fore-limb, failed to reveal any cause for the same and I again ordered him to my infirmary for treatment. On a second examination, after watching the animal for twenty-four hours, I diagnosed a paralysis of the entire limb, and suspected a central cause for the same. At this time the eye pit was in good condition, but in the face of a good appetite the animal had lost much flesh and was very sluggish in all his movements. He grew worse from day to day, when, on the 10th of September, I obtained the owner's consent to destroy him. Carefully removing his integument in the neighborhood of the face and fore limbs, I failed to find anything. I ordered the attendant to boil out the head and body and fore limbs. This revealed the presence of a misshapen rifle bullet at the anterior portion of the brain, about the point of emergence of the optic nerves, firmly imbedded in the nerve and brain tissue.

The meeting adjourned.

W. S. Kooker, *Secretary.*

